# CCOUC Ethnic Minority Health Project: A Case Study for Health EDRM Initiatives to Improve Disaster Preparedness in a Rural Chinese Population

**DOI:** 10.3390/ijerph18105322

**Published:** 2021-05-17

**Authors:** Kevin K. C. Hung, Makiko K. MacDermot, Emily Y. Y. Chan, Sida Liu, Zhe Huang, Chi S. Wong, Joseph H. Walline, Colin A. Graham

**Affiliations:** 1 Collaborating Centre for Oxford University and CUHK for Disaster and Medical Humanitarian Response (CCOUC), The Chinese University of Hong Kong, Hong Kong, China; kevin.hung@cuhk.edu.hk (K.K.C.H.); emily.chan@cuhk.edu.hk (E.Y.Y.C.); huangzhe@cuhk.edu.hk (Z.H.); cswong@cuhk.edu.hk (C.S.W.); 2 Accident and Emergency Medicine Academic Unit, The Chinese University of Hong Kong, Hong Kong, China; makikokato@cuhk.edu.hk (M.K.M.); jwallinemd@gmail.com (J.H.W.); 3 GX Foundation, Hong Kong, China; sida.liu@gxfoundation.hk

**Keywords:** disasters, health emergency and disaster risk management (Health EDRM), community-based intervention, disaster education, ethnic minority, China, community disaster resilience, Health EDRM workforce development

## Abstract

Disasters disproportionately impact poor and marginalised populations due to greater vulnerability induced by various risk determinants, such as compromised living conditions, language barriers, and limited resources for disaster risk management. Health Emergency and Disaster Risk Management (Health EDRM) emphasises a people- and community-centred approach for building stronger capacities in communities and countries since community members are often the first responders to health emergencies and should be central to effective risk management. A key action for promoting community disaster preparedness is the provision of Health EDRM education interventions. The Ethnic Minority Health Project (EHMP) has provided community-based Health EDRM education interventions in 16 ethnic minority-based villages in remote areas of China since 2009. It aims to enhance community disaster preparedness and resilience by improving health-risk literacy and self-help capacity at the individual and household levels. This case study outlines the first EHMP project in an ethnic minority-based community (Ma’an Qiao Village) in Sichuan Province, China. It highlights the key elements for planning and managing such a project and is a good demonstration of an effective Health EDRM workforce development project in rural communities. This report concludes with five recommendations for setting up a sustainable and effective Health EDRM education intervention in similar contexts.

## 1. Introduction

The health and socioeconomic impacts of disasters are influenced by the complex interactions between the severity and frequency of a hazard, the numbers of people exposed to the hazard, their vulnerability, and risk management capacities [1]. Many communities in the world are threatened by risks and consequences of health emergencies and disasters. Disasters can disproportionately impact certain groups in society, such as poor and marginalised populations, due to higher vulnerability and low resilience derived from underlying risk determinants, including poor living conditions, living in remote and hazard-prone areas, having fewer resources for disaster preparedness, and potential communication challenges with government agencies [2].

### 1.1. Improving Disaster Preparedness and Resilience in Communities

Health Emergency and Disaster Risk Management (Health EDRM) is an academic and policy paradigm, aiming to encourage research and activities to build stronger capacities in communities and countries to minimise vulnerability from disasters and consequent health risks [3]. Health EDRM emphasises a ‘people- and community-centred approach’ as community members are often the first responders to health emergencies and should be central to effective risk management.

One key element for the promotion of community disaster preparedness is the provision of Health EDRM education interventions in communities [3]. ‘Bottom-up’ health education initiatives originate from members of the community and promote community participation and empowerment by improving peoples’ awareness and knowledge of disaster health risks to strengthen their capacity-building behaviours to mitigate, prepare for, respond to, and recover from emergencies [4]. Designing and facilitating effective health education interventions is a complex process, involving planning, financing, coordination, implementation, and evaluation. There is limited evidence in the currently available literature on disaster preparedness efforts in ethnic minorities in rural China. This report constitutes one of the case studies in the WHO-funded project “Health workforce development strategy in Health EDRM: evidence from literature review, case studies and expert consultations” [5]. The aims of this case study report are to illustrate the EMHP as a Health EDRM workforce initiative by recognising people in communities as an important Health EDRM workforce group and to identify strategic recommendations that could be useful for developing Health EDRM workforce initiatives in similar contexts.

### 1.2. The Ethnic Minority Health Project

China is one of the most disaster-prone countries in the world, with the highest numbers of disasters reported between 2000 and 2019 [6]. It had a total population of 1.39 billion people in 2018, with 56 officially recognised ethnic groups [7]. Other than the majority Han Chinese ethnicity, the remaining 55 non-Han ethnic minority groups account for 8.48% of the total population [8]. Members of ethnic minority groups tend to live in remote, disaster-prone areas in rural Western or Northern regions in China and have lower incomes than Han Chinese groups [9]. The living standard of many rural ethnic minority-based communities has improved in the last decade due to rapid social and economic growth in China [7,10].

Nevertheless, ethnic minorities living in remote areas still have limited coping capacities for disasters derived from many risk drivers, including low levels of disaster preparedness due to limited resources, low educational attainment, language barriers to understanding the official language (Mandarin Chinese), and logistical challenges to receiving timely external assistance. However, a higher underlining vulnerability is not necessarily associated with higher risk awareness and a recent survey showed that only 20–30% of ethnic minority villagers felt prepared for disasters [11].

The EMHP was launched in 2009 and implemented community-based Health EDRM related education programmes in 16 rural Chinese villages across eight provinces [12]. The EMHP also took a ‘bottom-up’ approach, aiming to enhance community disaster preparedness by listening to and interacting with individuals and households in remote and rural communities to improve their health-risk literacy, disaster resilience, and self-help capacity [13].

This paper reviews the first EMHP programme conducted in an ethnic minority-based community (Ma’an Qiao Village) in Sichuan Province, China. It outlines the key elements for developing and managing such a project, which serves as an example of a community-based Health EDRM workforce development project. Based on the experience of these EMHP projects, this paper also formulates recommendations for setting up an effective and sustainable Health EDRM education intervention for communities in rural and/or ethnic minority villages.

## 2. The Ma’an Qiao Village Programme

An EMHP programme was implemented in Ma’an Qiao Village during 2009–2011 after the Panzhihua earthquake in 2008. The same village was revisited in 2018 for a long-term evaluation.

Ma’an Qiao Village is located in the Jinsha River area of the Xin’an Township, Huili County, Liangshan Yi Autonomous Prefecture, Sichuan Province in Southwest China and is populated by both Dai and Yi ethnic minorities. It is composed of over 1200 villagers and 300 households in seven sub-villages [11]. The Panzhihua earthquake in August 2008 destroyed large portions of infrastructure in this village. Nevertheless, the village did not receive timely and adequate support due to minimal external help and media coverage.

The project team first explored the village in 2009 and selected it as the first EHMP project site, based on the four selection criteria; (i) geographical remoteness; (ii) ethnic minority representation; (iii) economic vulnerability (living on under USD 1.25/person/day); and (iv) disaster prone-ness [5,14].

### 2.1. Planning

**Figure 1:**
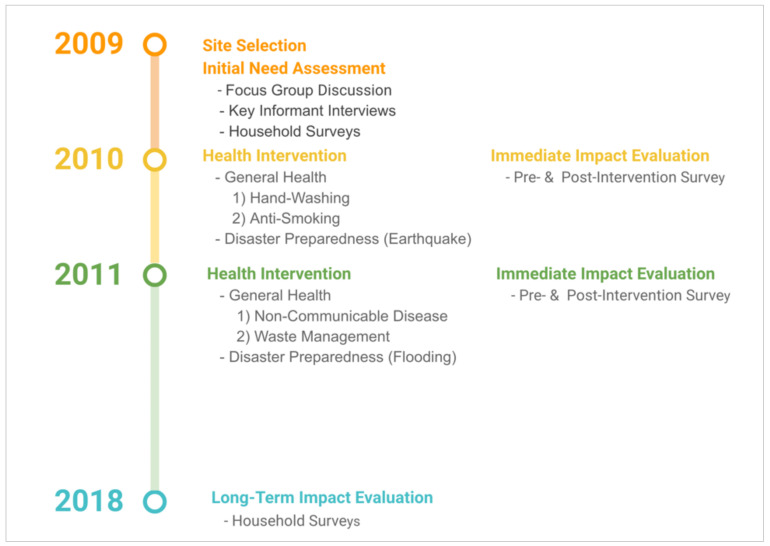
Project Timeline and Outline for the Ma’an Qiao Village Programme.

Following the MAP-IT programme planning model [15], the Ma’an Qiao Village programme involved four key steps of programme management: needs assessment, planning, intervention implementation, and outcome evaluation (immediate and long-term). The timeline and outline for the Ma’an Qiao Village programme is illustrated in Figure 1.

A team, composed of public health practitioners and student volunteers, was deployed to the village in 2009, 2010, and 2011. Lack of baseline data is common in remote villages in China. Thus, a needs assessment was conducted through surveys, interviews and focus group discussions with local stakeholders to capture the evolving health needs and priorities for evidence-based programme planning. Discussions with village leaders confirmed that no health interventions had been introduced to the village previously.

Based on the 2009 and 2010 needs assessments, the most suitable interventions with specific objectives and outcomes were developed and implemented in 2010 and 2011. The 2009 needs assessment indicated the immediate health concerns, such as a safe living environment, access to healthcare, infectious disease risks arising from poor hygiene practices and health risks from earthquakes.

The 2010 needs assessment, 18 months post-disaster, indicated a shift in primary health concerns toward longer-term health issues such as waste management and non-communicable diseases (NCDs). Disaster preparedness for future floods was also concerned as the village had a long history of recurrent floods.

### 2.2. Multidisciplinary Approach

The Ma’an Qiao Village programme was initiated and managed in a collaborative effort between a university, a non-governmental organization (NGO), local governmental bodies, and local stakeholders.

The Wu Zhi Qiao Charitable Foundation (WZQ) is an academic-architectural community initiative based in Hong Kong specialising in supporting sustainable development in Chinese villages. WZQ was the NGO stakeholder for this project and mediated communications between local stakeholders and the project team from CCOUC. Local governmental bodies were involved in identifying disaster high-risk areas and providing data about the village and useful contacts. In-depth discussions with community leaders provided specific data about the village, such as hazards, risks, health-related behaviours and culture. Throughout the programme, key persons in the community were also important to create a rapport between villagers and the team members delivering the project. Student volunteers promoted health intervention activities and helped conduct surveys. All key stakeholders’ expertise and responsibilities are listed in Table 1.

## 3. Interventions

Health EDRM emphasises a “systematic approach that takes account of the risks, capacities, and the availability of resources to implement risk management measures at the local, subnational and national levels” [3]. The establishment of the community capacity for health EDRM is one of the key components in the WHO Health EDRM Framework [3]. The health education interventions were delivered in 2010 and 2011 to 110 villagers and 125 villagers respectively.

### 3.1. Health Education Topics

Based on the findings from the 2009 and 2010 needs assessments, Health EDRM education interventions in Ma’an Qiao Village gave priority to five education topics: (1) hand hygiene; (2) anti-smoking behaviour; (3) non-communicable disease management; (4) waste management; and (5) disaster preparedness. For each intervention, specific goals, activities and outcome measures were predetermined.

Target participants were adult household representatives (≥18 years old). The majority of residents in these villagers were retired elderly people and children with limited education levels. After discussions with community leaders, language differences were flagged as a potential barrier for delivering health education messages to villagers since many residents faced language barriers to understanding the official language (Mandarin). Therefore, the project team decided to use a combination of participatory approaches, such as workshops and group discussions, and pictorial presentations, e.g., posters and drama performances, to increase participants’ understanding and engagement [13,16,17].

#### 3.1.1. Hand Hygiene

This intervention aimed to teach the importance of good hand-washing practice in order to reduce the health risks of contracting infectious diseases. Activities involved demonstrating when and how to wash hands whilst circulating five sets of pictures depicting the steps of handwashing among participants.

#### 3.1.2. Smoking Behaviour

Anti-smoking sessions were originally aimed at motivating people to quit smoking. However, it was realised that this was an unrealistic immediate goal for the community since smoking was strongly connected to their culture and beliefs, e.g., associated with masculinity; this would be hard to change by a one-off intervention from a non-local education team. Therefore, the objective was modified to increase awareness of adverse health risks from smoking and passive smoking, e.g., lung cancer, heart diseases, and chronic obstructive pulmonary disease (COPD). Group discussions were conducted to discuss the relationship between smoking habits and potential health risks.

#### 3.1.3. Non-Communicable Disease Management

This intervention was to increase awareness of healthy/unhealthy diets and risky behaviours, and their influence on non-communicable diseases (NCDs) such as hypertension and diabetes. The activity delivered three key messages; (i) risk factors for unhealthy outcomes; (ii) better dietary habits for healthy outcomes; and (iii) the way to adopt healthy behaviours. Examples of healthy/unhealthy food were presented using posters. Messages illustrating health risks of alcohol were conveyed via drama in a comical fashion to gain local people’s attention and engagement.

#### 3.1.4. Waste Management

The waste management intervention aimed to enhance awareness and knowledge in (i) health risks related to improper waste management and (ii) possible solutions to reduce the risks. The messages were delivered using posters. A poster illustrated the causal relationship between mishandled waste (e.g., kitchen waste or excreta), vectors (e.g., flies), and illness (e.g., diarrhoea) to highlight the potential health risks from improper waste management. Another poster described the solutions to avoiding potential illness by indicating proper disposal methods, leading to a clean environment and good health outcomes.

#### 3.1.5. Disaster Preparedness

The disaster preparedness interventions were to improve awareness, beliefs and self-help capacity in the immediate response and preparedness for future earthquakes and floods. The activity was divided into three parts. The first part focused on the pre-disaster phase by demonstrating how to prepare and use basic lifesaving items, such as fire-starters, a whistle, a flashlight, a multi-purpose pocket knife, and a recipe for an oral rehydration solution (printed on a grab bag). A bag with all these items was given to each participant at the end of the activity as a reminder and souvenir.

The second part demonstrated basic survival and evacuation skills during earthquakes or floods, for example, how to survive under earthquake rubble or how to stay away from floodwaters and power lines. The third part described how to avoid health risks in the post-disaster phase, such as secondary injuries from damaged houses and diseases caused by contaminated water.

### 3.2. Outcome Evaluation

The effectiveness of each health education intervention was assessed by comparing the results of a questionnaire before and immediately after interventions in 2010 and 2011. The village was revisited for the long-term outcome evaluation in 2018. Survey participants in 2010, 2011, and 2018, were adult household representatives over 18 years old. This project employed convenience sampling: participants were selected based on their availability and willingness to take part. Snowball sampling methods were also used to increase sample size by asking existing participants to nominate more people known to them in the same village. Villagers were invited to join the interventions through promotional activities such as household visits, leaflet distribution, and posters. Participants were then asked to complete pre- and post-intervention surveys at the intervention site. Four local dialect translators were recruited to facilitate communication as a language barrier between the local dialect and Mandarin was expected for a high percentage of villagers. The characteristics of participants in the three interventions and surveys are summarised in Table 2.

#### 3.2.1. Immediate Outcome Evaluation

Pre- and post-intervention cross sectional surveys were administered to participants to evaluate the effectiveness by assessing predetermined outcome measures: improvements in knowledge and beliefs were considered to indicate subjective level of preparedness as a proxy for possible future behavioural change for better disaster preparedness [4,11].

Overall, the 2010 & 2011 Health EDRM education interventions improved their knowledge in health risks in relation to personal habits, environment and disasters immediately after the interventions. Furthermore, the respondents in Ma’an Qiao community increased confidence in managing their disaster risks and understood the importance of disaster preparedness. The results of outcome evaluation are summarised in Table 3. Comparison between pre & post groups was performed using the Pearson Chi-Square test (***χ*^2^** tests) and a *p* value of <0.05 was considered statistically significant.

#### 3.2.2. Long-Term Outcome Evaluation

The 2018 long-term outcome evaluation indicated long-term knowledge retention in handwashing practice and anti-smoking beliefs. However, reduced knowledge was found in knowledge on NCD management and waste management. The confidence to deal with disasters and intention to prepare disaster kits also dropped in 2018. These results confirm the importance of continuity and sustainability of health education intervention provision to maintain knowledge of and beliefs in healthy behaviours for the longer term.

The 2018 survey also assessed health-related lifestyle changes in the village. It indicated dramatic lifestyle shifts in the villagers since 2011, particularly in health status, education, income levels, and the use of modern technology. 70% of respondents reported having access to the Internet and having an average of 2.6 mobile phones per household in 2018, whereas only 20% of the village households had some access to mobile telecommunication devices in 2010. Furthermore, over 35% of households reported in 2018 that they would use the internet to obtain disaster-related information and more than 60% of them would install a disaster-related mobile application [4,14]. Furthermore, many villagers had migrated to urban areas for work, which necessitated a substantial increase in the use of mobile phones and other digital communication devices to communicate with their family members.

## 4. The EMHP: Achievements and Scaling-Up

### 4.1. Project Outcome

The EMHP community-based Health EDRM education programmes were implemented in 16 ethnic minority-based rural villages across eight provinces in China [14]. A series of training manuals and action guidelines for planning and implementing interventions were developed based on the experience from each programme [18].

The project also provided learning opportunities for an academic institution, governmental and non-governmental bodies, and local communities to work together to achieve shared goals in Health EDRM. Student volunteers also gained valuable field experience in Health EDRM education which could be useful for future programme planning. The findings from the EMHP have been shared with all partners and published in several international public health and disaster-related journals [4,11,14].

### 4.2. Scaling-Up

Rapid socioeconomic growth has been observed everywhere in China. The situation analysis in 2018 by the EMHP project team indicated the prevalence of a more modernised lifestyle in rural communities in China, which also altered the health needs and priorities of these communities. The 2018 survey also highlighted an increased willingness among rural communities to receive Health EDRM information through mobile technology, suggesting a need to change risk communication strategies for health education interventions for these communities. Therefore, the project team decided to scale up the EHMP to provide Health EDRM education online and is currently developing a Health EDRM education mobile app to be used in ethnic minority-based villages in China.

## 5. Limitations

Firstly, although a single case study can give us deeper understanding of a single instance or a phenomenon, the generalisability and transferability of the findings to a wider population is less assured. Secondly, validity and reliability of a case study by its very nature remains a challenge. Therefore, the findings from this case study should be carefully translated in each unique context with its different population needs. Thirdly, evaluating and measuring the true impacts of disaster education/promotion initiatives in the context of disaster risk reduction has inherent challenges as these evaluations are subject to a disaster occurring [19]. Lastly, pre and post cross-sectional surveys were used to measure changes in self-reported beliefs or levels of understanding. Self-reported measures have less validity as participants may have exaggerated their understanding or may have under-reported as they did not understand the content or forgot certain details. In order to maximise the verbal understanding of the intervention, local dialect interpreters were always employed.

## 6. Recommendations

The findings indicated that the EMHP program produced positive outcomes which may suggest the increased capacities of the target communities to prepare for future hazards and to respond in case of an emergency at both household and community levels.

Despite the limitations of a single case study, the EMHP program should serve as a good example when building a bottom-up intervention for Health-EDRM at the community level. Based on these experiences and lessons, the following five recommendations are created to contribute to the Health EDRM health workforce research project for developing effective and sustainable community-based Health EDRM education initiatives focusing on individuals and communities in rural settings. These recommendations could also be framed as a working hypothesis of potential community health disaster risk reduction impact for future studies.

Firstly, we recommend an evidence-based ‘bottom-up’ approach through strategic planning with clear and measurable outcomes when developing a Health EDRM intervention to empower people as potential first responders and Health EDRM workforce in rural communities. From the EMHP experience, the ‘bottom-up’ approach is effective in developing evidence-based interventions for specific local communities by identifying local hazards, vulnerabilities, and underlying risk determinants. This specifies actual needs and priorities in the target community and minimises the gap between what is needed and what is provided [20,21,22].

However, developing and delivering sustainable community-based interventions is challenging in rural areas due to limited resources, capacity, and weak public health systems. To effectively plan and implement a community-based intervention, the Ma’an Qiao Village project highlighted the importance of taking a step-by-step approach following the project cycle management. A project cycle requires strategic planning by structuring a programme management into key stages, e.g., planning, implementation, and evaluation [23,24].

At the planning stage, based on the needs assessments, relevant and achievable goals and outcomes are predetermined and the activities are planned to achieve them. This is also important for evaluation the effectiveness. However, common to many public health initiatives, measuring intervention effectiveness was a challenge for the EMHP. This is due to various reasons.

First of all, the rapid changes in lifestyle and demography in remote villages in China also resulted in changes in their health needs and priorities. Secondly, the project outcomes might be influenced by many external factors, e.g., accessing information online as an alternative cause of changing knowledge and attitude. Thirdly, it was hard to assess the project’s outcome over the project lifetime given people migrating in and out of the programme catchment area. Fourthly, due to language barriers and cultural differences, it could be difficult to estimate participants’ level of understanding even with translators. Lastly, much of what was measured were participants’ intentions to implement improved disaster preparedness behaviours, and it is unclear whether that was translated into actual behavioural change.

Therefore, outcome indicators needed to be concise, measurable and achievable and to be revised for each new programme [4]. The evaluations and continuous needs assessment provided important information for reshaping the programme in response to short- or long-term changes in needs or priorities in the target community. These re-evaluations also contribute to relevance, efficiency, continuity, and sustainability of community-based interventions [25].

Our second recommendation is to facilitate multidisciplinary partnerships and active community involvement from the planning stage of community-based Health EDRM workforce development programmes. The EHMP benefited greatly from the active participation of a wide range of stakeholders from all levels of society from the beginning. Their roles, responsibilities, levels of engagement, contributions, and decision-making structures were clarified, supported, and resourced in order to build and maintain participatory and transparent relationships. Documenting and sharing information on progress and findings with all partners also encouraged the transparency and sustainability of the project. In the EMHP, proactive engagement with local stakeholders, such as village leaders and local health workers, was key for the bottom-up initiatives to remain context-specific, culturally appropriate, efficient, and cost-effective. The community members were always actively involved from planning till evaluation. Depending on the community, the participating partners may vary; however, all partners must have a common understanding of problems and share the same visions and goals [26,27,28].

Building a long-term relationship with a target community can support better programme follow-up, continuous quality data for research, and may create an opportunity to hand over ownership of the project to the community [22]. Multidisciplinary partnerships are also essential for successful ‘bottom-up’ initiatives as they can yield greater impact by combining the expertise of every project stakeholder [29,30,31]. Financial, administrative, and policy making inputs from central and local government bodies could help integrate community-based Health EDRM education initiatives into other local and national government programmes for longer sustainability.

Civil society and community-based organisations can play significant roles in supporting and advocating for marginalised groups and individuals [32]. For instance, the Ma’an Qiao Village programme team made first contact with a village person through a locally recognised NGO (i.e., WZQ). The WZQ did not only help to mediate local connections, but they also contributed to the high turn-out in the interventions as some villagers joined the interventions primarily to learn about the WZQ’s plan for building infrastructure. These characteristics from the EMHP demonstrated the importance of stakeholder engagements.

Thirdly, we recommend providing community-based Health EDRM education interventions with specific local information. Disaster education programmes positively influence safety awareness, readiness level, and the establishment of individual self-help capacity [33]. A low level of awareness and understanding of health risks was negatively correlated with readiness for disasters and preparing personal protective measures [34]. Furthermore, disaster education programmes which primarily provide generic information on disaster preparedness had fewer impacts on behavioural changes [35].

Since standardised disaster information is readily available at national, sub-national, and local levels, if the generic information is to be adopted, it should be modified to fit local contexts before use since these programmes have higher impacts when people can relate their own environmental hazards and health risks to the topics [35,36]. In the case of the EHMP, based on the healthy community-planning framework from the ‘Healthy Village’ programme under the World Health Organization [11,37], five key education topics were developed, which are relevant to local health concerns in rural villages in China: water and sanitation, indoor air environment, waste management, non-communicable disease management, and disaster preparedness. For each programme and related interventions in a different village, these topics were polished to fit into more local specific contents based on needs assessments.

The needs assessments were vital for identifying local hazard-related profiles (e.g., local disaster knowledge, community emergency management systems, current and future hazard risks), and community profiles (e.g., baseline health status, demography, cultural/education background, and health beliefs). Discussion with local leaders and local health workers also helped identify contextual needs and priorities.

Furthermore, disaster education programmes may benefit through hands-on experience as people retain knowledge better through active participation in the learning experience [36,38,39,40,41]. An active learning approach was used in the EHMP to teach villagers how to use life-saving items and what to be included in a disaster grab bag. It promoted the knowledge of what each item is/does as well as confidence in using it [42]. These were the keys to the success of the EMHP.

Fourthly, we recommend mapping all necessary financial and human resources before designing a community-based Health EDRM education intervention to maximise the effectiveness. Stable finance, established institutional capacity, human resources and good coordination mechanism were essential elements in the effectiveness and sustainability of the EMHP. The results of all field trips were reported to funders or donors to maintain trust, transparency and programme sustainability. Strong leadership and having experienced multidisciplinary team members for planning, implementation, and evaluation were also key in the EHMP. The team had experienced members of various backgrounds and capabilities who had a good understanding of local culture, and those with good experience in organising fieldwork expeditions and logistics, e.g., transportation, lodging, and meals.

To conduct the interventions efficiently, detailed briefings and timetables were arranged before any intervention trips. Availability of clear step-by-step guidelines was useful for team coordination, particularly when involving inexperienced staff and volunteers. These instructions included contingency plans as some interventions needed to be modified in real time during the trips.

Finally, we recommend considering the use of digital information technologies, such as mobile phone applications (‘mobile apps’) and social media, for future community-based Health EDRM initiatives. Mobile phones have become pervasive communication tools worldwide due to their affordability, simplicity, and accessibility. Earlier data indicated that more than 80% of the world’s population had mobile phones and 8 in 10 individuals in developing countries owned a phone [43,44]. In China, around 30% of internet users in 2020 were from rural areas [45]. The 2018 EHMP situation analysis identified the increasing availability and popularity of internet and mobile phone use in the remote villages in China, particularly among the younger and educated villagers.

Approximately 60% of participants showed interest in installing a mobile app to learn disaster preparedness [4]. Based on this evidence, the EMHP has been scaled up to develop a mobile app to deliver Health EDRM-related messages to rural communities in China. The widespread prevalence of mobile technologies expands opportunities for Mobile Health (mHealth) for Health EDRM education initiatives, particularly in low- and middle-income countries and for people in remote villages or marginalised population groups [46,47,48]. 

Increasing use of digital technology could be a cost-efficient approach for disseminating consistent and timely disaster information to both general and at-risk populations [49,50]. Although the effectiveness needs more research, various mobile apps for disaster education to influence health-related behaviours have been developed [51,52]. For example, an app targeting elementary school children in Indonesia was found to increase their understanding of earthquake disaster responses [53]. The *Disaster Preparedness Tokyo* app, developed by the Tokyo Metropolitan Government in Japan, provides generic knowledge on disaster preparedness and response in multiple languages, using various formats, such as disaster maps, quizzes, real-time alerts and evacuation advisories in extreme events to help people make timely life-saving decisions [54].

Similarly, social media tools, such as Facebook, YouTube, Twitter and WeChat, have been widely used recently for all phases of disaster risk management [44]. Social media can disseminate accurate and science-based health information to a diverse audience and facilitate information exchange and behavioural change [49]. Building on these functionalities, there are various social media platforms available to increase disaster awareness and preparedness for a range of hazards including tsunamis, earthquakes, flood, and wildfires [35].

However, social media use should not be framed as a universal risk communication channel since relying on the use of social media could reduce access to information in some population groups who are not technologically connected, such as the elderly or people without internet access. Furthermore, challenges associated with social media use, such as managing misinformation and ensuring user privacy protections, also need to be taken into account at the planning stage [49,55].

## 7. Conclusions

Developing and delivering evidence-based, effective, and sustainable community-based Health EDRM education interventions is challenging in rural areas and in developing countries due to limited resources and capacities, and weak public health systems.

The Ma’an Qiao EMHP programme demonstrated a pathway for developing and managing evidence-based Health EDRM education interventions to increase disaster resilience at the community level by changing people’s beliefs and behaviours. This case study highlighted how developing and managing such a project is very complex and requires strategic planning for maximising the project’s effectiveness and sustainability from the start.

This project provides an example to highlight the importance and success of disaster risk reduction in ethnic minority rural communities. Five strategic recommendations are offered for consideration when planning a project in similar context, focusing on community-based Health EDRM workforce development: (i) community-based interventions through a ‘bottom-up’ approach, (ii) multidisciplinary partnerships, (iii) selection of locally relevant Health EDRM education topics, (iv) advance planning for all necessary resources, and (v) use of digital information technology. With these recommendations, future Health EDRM education projects may stand a higher chance of achieving their goals.

## Figures and Tables

**Table 1:** Key Stakeholders and their Responsibilities.

Stakeholders	Sectors	Responsibilities
CCOUC (Physicians and public health professionals)	Public Health/ Medicine/ Academic and Research	Health needs assessment Planning, implementing and evaluating the programme Recruiting university student volunteers Planning and managing village trips
The Wu Zhi Qiao Charitable Foundation	Architecture/ Housing	Needs assessment for sustainable development, e.g., footbridges and facilities Facilitating access to communities Sharing local knowledge based on previous work in the village
Local Government	Government	Sharing information/data about the village Facilitating community access
Village Leaders	Local Stakeholders	Promoting and supporting activities throughout interventions Facilitating rapport between villagers and programme implementors
University Student Volunteers	Tertiary Education	Manpower Facilitating promotion activities Conducting pre- and post-intervention evaluation surveys

**Table 2:** Characteristics of Participants in the 2010, 2011 and 2018 Surveys in the Ma’an Qiao Village Programme.

	2010 ^a^ (*n* = 110)	2011 ^b^ (*n* = 171)	2018 ^c^ (*n* = 71)
Male to female ratio	1:1.6	1:1.3	1:1.4
Age Category (years)			
<45	Mean = 44.5 Range = 20 to 73 >40 = 58%	49.7%	42.3%
45–59		26.3%	31.0%
≥60		24.0%	26.8%
Educational Attainment			
Non-Literate	31%	41.0%	24.0%
Primary School	53%	43.3%	46.5%
Junior High School	NA	12.3%	21.1%
Senior High School	NA	2.9%	5.6%
Tertiary School	NA	0.6%	2.8%
Mean Household Size	NA	3.9	4.2
Income per person (RMB)	NA	4029	18,026
Agricultural Sector Occupation	83%	88.8%	94.4%
Self-Reported Health Status			
Good	NA	39.5%	61.8%
Average	NA	NA	16.2%
Poor	NA	NA	22.1%

Data Sources: ^a^ Data collected during the 2010 visit; ^b^ Data collected during the 2011 visit; ^c^ Data collected during the 2018 visit.

**Table 3:** Changes in beliefs and knowledge in the 2010/2011 pre- and post-intervention and the 2018 evaluation survey.

Types (No. of Respondents)	Outcome Measures Health-Related Beliefs and Knowledge	2010 & 2011 Interventions		2018
		Before	After	Evaluation
**Hand hygiene** (*n* = 100)	Believing in the need of handwashing before meal	88%	99%	97%
	Believing in the need of handwashing after toilet	90%	93%	93%
	Knowing the health risks (e.g., diarrhoea) of poor hand hygiene	85%	99% (14%↑ *p* < 0.01)	87% (12%↓ *p* < 0.01)
**Anti-smoking** (*n* = 98)	Believing that smoking can cause lung cancer	64%	94%	94%
	Believing that passive smoking is more harmful for children	80%	97%	91%
**Waste management** (*n* = 125)	Knowing the health risks of misplaced excreta	84%	99% (15%↑ *p* < 0.01)	-
	Knowing health risks of misplaced kitchen waste	81%	96% (15%↑ *p* < 0.01)	-
	Believing that it is safe to burn different types of wastes together	67%	47% (20%↓ *p* < 0.01)	47%
**Non-communicable diseases management** (*n* = 125)	Knowledge of hypertension	57%	85% (28%↑ *p* < 0.001)	-
	Knowing the health risks (hypertension) of high salt intake	71%	95% (24%↑ *p* < 0.001)	81% (14%↓ *p* < 0.01)
	Knowing health risks (diabetics) of higher meat intake	41%	79% (35%↑ *p* < 0.001)	-
**Disaster Preparedness** (*n* = 110)	Believing the importance of disaster preparedness	88%	95%	93%
	Knowing how to prepare a ‘disaster kit’	45%	65% (20%↑ *p* < 0.05)	-
	Intention to prepare a ‘disaster kit’	58%	93% (35%↑ *p* < 0.001)	68% (27%↓ *p* < 0.01)
	Being confident in dealing with future disasters	89%	95%	85% (10%↓ *p* < 0.05)
	Knowing how to evacuate from floods	94%	98%	98%

***χ*^2^** tests were conducted between the percentage of 2010–2011 pre- and post-intervention results and between 2010/2011 post-intervention and 2018 evaluation results. ↑ = increase, ↓ = decrease.

## Data Availability

The datasets generated during the current study are available from the corresponding author on reasonable request.
